# Matrix Isolation and Solvation of the Benzonitrile Radical Anion

**DOI:** 10.1002/chem.202501150

**Published:** 2025-05-30

**Authors:** Shubhra Sarkar, Ankit Somani, Wolfram Sander

**Affiliations:** ^1^ Lehrstuhl für Organische Chemie II, Ruhr‐Universität Bochum 44801 Bochum Germany

**Keywords:** electron transfer, microhydration, radical anion, reactive intermediate, solvation

## Abstract

Solvated electrons, one of the strongest reducing agents, exhibit short lifetimes in the range of pico‐ to milliseconds when generated photochemically or by radiolysis in solution. In contrast, solvated electrons produced using sodium metal in liquid ammonia are stable for days and have long been used in synthetic chemistry. Using sodium atoms as an electron source, we were able to trap solvated electrons in low‐density amorphous (LDA) water ice matrices with lifetimes of several days and use these electrons as reducing reagents. In LDA matrices doped with benzonitrile **1,** these electrons react with **1** to form the benzonitrile radical anion **2**. In argon matrices, in the absence of water, only small amounts of **2** were observed after deposition, and most of the sodium and **1** remained unreacted. The yield of radical anion **2** is significantly higher in amorphous water ice than in solid argon. The photoexcitation of radical anion **2** in both argon and LDA water ice matrices resulted in a reversal of the electron transfer under back formation of benzonitrile **1**. Annealing of argon matrices doped with small amounts of water containing **2** resulted in the formation of 1:1 and 2:1 hydrogen‐bonded complexes between water and radical anion **2**.

## Graphical abstract

Hydrated electrons are potent reducing agents that may play significant roles in chemical evolution within astrochemical and prebiotic environments. Benzonitrile, a molecule present in the interstellar medium (ISM), can capture electrons in amorphous water ice at cryogenic temperatures, forming a radical anion. This radical anion engages in hydrogen bonding with surrounding water molecules, interactions that can be characterized using infrared (IR) spectroscopy.

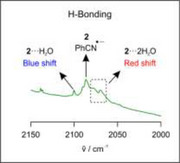

## 1 Introduction

Benzonitrile **1** is the simplest nitrogen‐bearing aromatic molecule discovered in the interstellar medium (ISM). It was observed in the core of the starless cloud TMC‐1^[^ ^1^ ^]^ and presumably is a precursor of more complex nitrogen‐containing polyaromatic hydrocarbons. However, the reaction pathway for the formation of **1** in the ISM is still unknown. Henceforth, the gas phase chemistry of **1** has become a focus of research.^[^ ^2^, ^3^, ^4^, ^5^ ^]^ Benzonitrile **1** has a large dipole moment (> 4 Debye) facilitating its detection through radio astronomy. Electron addition to **1** results in the formation of the benzonitrile radical anion **2**, which, given the abundance of free electrons in the ISM, could be a missing link in reactions under astrochemical conditions of **1**.

**Scheme 1:**
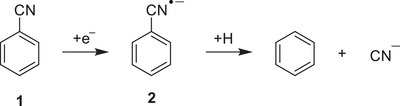
Addition of an electron to **1** to generate **2** and subsequent decomposition into benzene and CN^¯^.

Vecera and coauthors recently reported a significant breakthrough in simplifying industrial graphene production using benzonitrile.^[^ ^6^ ^]^ Benzonitrile enables the quantitative discharge of reduced graphite forms, including graphite intercalation compounds and graphenides, onto surfaces. With its low reduction potential, benzonitrile forms a radical anion that aids in measuring negative charges on carbon sheets. This enhancement of the chemical exfoliation method leads to the production of defect‐free graphene layers with controllable conductivity. In solution, the radical anion **2** and several of its derivatives were synthesized by electrochemical reduction or by electron bombardment of the neutral nitriles.^[^ ^7^, ^8^, ^9^, ^10^ ^]^ The radical anion generated through electrochemical reduction in anhydrous dimethylformamide (DMF) readily dissociates into the CN anion and the phenyl radical, which upon H‐abstraction from the solvent produces benzene (Scheme 1).^[^ ^11^ ^]^ Chutny and coworkers generated radical anion **2** by pulse radiolysis of an aqueous solution of **1** and characterized it by absorption spectroscopy.^[^ ^12^ ^]^ Hydrated electrons, hydrogen atoms, and hydroxyl radicals were produced in a pulse radiolysis experiment where hydrated electrons react with **1** to generate **2**. In acidic solution, H atom abstraction was observed. In the gas phase, the dissociative attachment of slow electrons (<17 eV) to a series of saturated nitriles was studied by Heni and Illenberger using mass spectrometry.^[^ ^13^ ^]^ A mono‐energetic electron beam was crossed with a molecular beam of nitrile molecules, and the negative anions were detected as a function of the electron energy using a quadrupole mass filter. However, **2** could not be observed in these experiments, and the authors concluded that the benzonitrile radical anion **2** might not be stable enough to be detected in the gas phase. Burrow et al. came to a similar conclusion using electron transmission spectroscopy (ETS).^[^ ^14^ ^]^ The authors explain that the ^2^B_1_ ground state of **2** is bound by only a few tenths of an electron volt, hence it was impossible to detect this radical anion in ETS.

Maeyama and coworkers generated the radical anion **2**, solvated by individual molecules of water or methanol, through the attachment of photoinduced electrons in a supersonic expansion of benzonitrile vapor seeded in neon gas and characterized using photodetachment efficiency spectroscopy.^[^ ^15^ ^]^ This study was limited to the spectroscopic range between 3000 and 4000 cm^−1^, and therefore the characteristic C≡N stretching mode could not be observed as it is expected to be around 2100 cm^−1^. Molecular clusters between **2** and several water molecules in the gas phase were also investigated by Dixon et al. using negative‐ion photoelectron spectroscopy (NIPES) and imaging.^[^ ^16^ ^]^ The ground state of the bare as well as the microsolvated radical anion **2** was experimentally found to be the valence ^2^B_1_ state. However, the spectral assignment was later challenged by Adamowicz et al., who claimed that such a state of **2** was “unphysical” and predicted a dipole‐bound ^2^A_1_ ground state.^[^ ^17^ ^]^ Finally, the electronic nature of the ground state of **2** was unraveled in a theoretical study by Krylov et al. using equation‐of‐motion coupled‐cluster theory.^[^ ^18^ ^]^ They confirmed the ^2^B_1_ ground state deduced from the NIPES experiments, however, the first excited state, the dipole bound ^2^A_1_ state, is only ∼0.1 eV (2 kcal/mol) higher in energy. The ^2^B_1_ ground state of **2** was predicted to be stabilized by interaction with a single water molecule.

Under the experimental conditions described above, the benzonitrile radical anion **2** is a short‐lived intermediate, which prevents its isolation and the study of its chemical reactivity. In this work, we describe a novel method to synthesize **2** via electron transfer from sodium atoms to benzonitrile **1** in cryogenic matrices. Since **2** is stable in these matrices, it could be characterized by IR, UV‐vis, and electron paramagnetic resonance (EPR), spectroscopy, and interactions of **2** with one and two water molecules could be studied.

## 2 Results and Discussion

### 2.1 Formation of Radical Anion 2 in Argon Matrices

**Figure 1:**
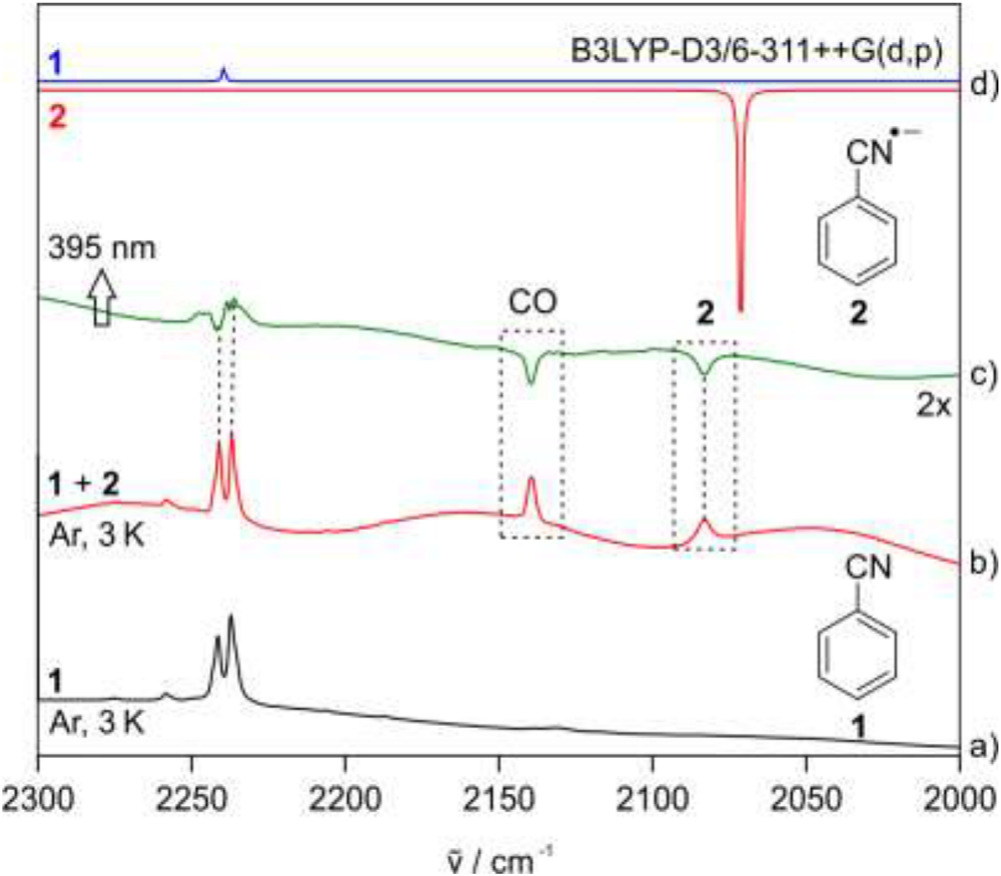
IR spectra showing the reaction between sodium and benzonitrile **1** in an argon matrix. a) IR spectrum of **1** at 3 K. b) IR spectrum between 2000 and 2300 cm^−1^ obtained after codeposition of sodium and **1**, leading to the formation of radical anion **2**. c) Difference IR spectrum after irradiating the matrix containing **1** and **2** with 395 nm light. The band pointing downward is assigned to the CN stretching vibration of **2** and the band pointing upward is assigned to the CN stretching vibration of **1**. d) Computed CN stretching vibration band of **1** (blue, peak pointing upward) and of radical anion **2** (red, peak pointing downward) at B3LYP‐D3/6–311G++(d,p) level of theory, and frequencies are scaled by a factor of 0.96.

Sodium and benzonitrile **1** were deposited together with a large excess of argon on a spectroscopic window at 3 K. IR spectra show the expected bands of **1** with the characteristic C≡N stretching vibrations at 2241 and 2237 cm^−^^1^. The splitting into a doublet is caused by matrix‐site effects as reported by Sundararajan and coworkers.^[^ ^19^ ^]^ Additionally, a new band is observed at 2083 cm^−1^ which is assigned to the C≡N stretching vibration of radical anion **2**. The IR bands of **2** are in good agreement with the literature data.^[^ ^15^, ^20^ ^]^ The large red shift of approximately 155 cm^−1^ of the CN stretching vibration of **2** compared to that of **1** indicates the weakening of the CN bond (Figure 1b, Table 1). The smaller intensity of the CN stretching vibration of **2** compared to that of **1** suggests that only a small fraction of **1** is converted into **2**. As expected, the yield of the radical anion **2** increases with increasing concentration of sodium atoms. The assignment of the IR spectrum of **2** was confirmed by isotopic labeling using [^13^C‐**1**] benzonitrile. The ^13^C isotopic shift of the CN stretching vibration of **2** was measured to be ∼48 cm^−1^, in good agreement with DFT calculations at the B3LYP‐D3/6–311++G (d,p) level of theory (Figure S2).

**Scheme 2:**
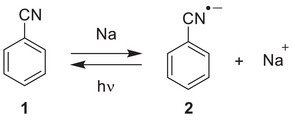
Reaction of sodium and benzonitrile **1** in argon matrices at 3 K.

Irradiation of the matrix with 395 nm leads to the disappearance of all bands of **2** and an increase in the intensity of the bands of **1**, indicating the photoionization of matrix‐isolated **2**. The excitation of the sodium atoms with visible light (535‐600 nm) also leads to the depletion of **2** instead of the expected increase of **2** by electron transfer from sodium atoms to **1** (Figure 1c, Scheme 2). This differs from our previous experiments studying the electron transfer to benzophenone, where irradiation of sodium atoms with visible light results in an increase of the benzophenone radical anion due to the electron transfer initiated by the excitation of sodium atoms.^[^ ^21^ ^]^ Due to the low electron affinity (EA) of **1**, which is 0.28 eV (6.4 kcal/mol) calculated at the B3LYP‐D3/6–311++G(d,p) level of theory, the electron transfer is not efficient, resulting in a low yield of **2**.^[^ ^18^ ^]^ Therefore, only the most intense IR bands of **2** could be observed (Figure S1). In addition to the bands of benzonitrile **1** and its radical anion **2**, a band at 2147 cm^−1^ is assigned to a CO impurity introduced during heating the Knudsen cell to higher temperatures.

### 2.2 Microhydration of Radical Anion 2

**Figure 2:**
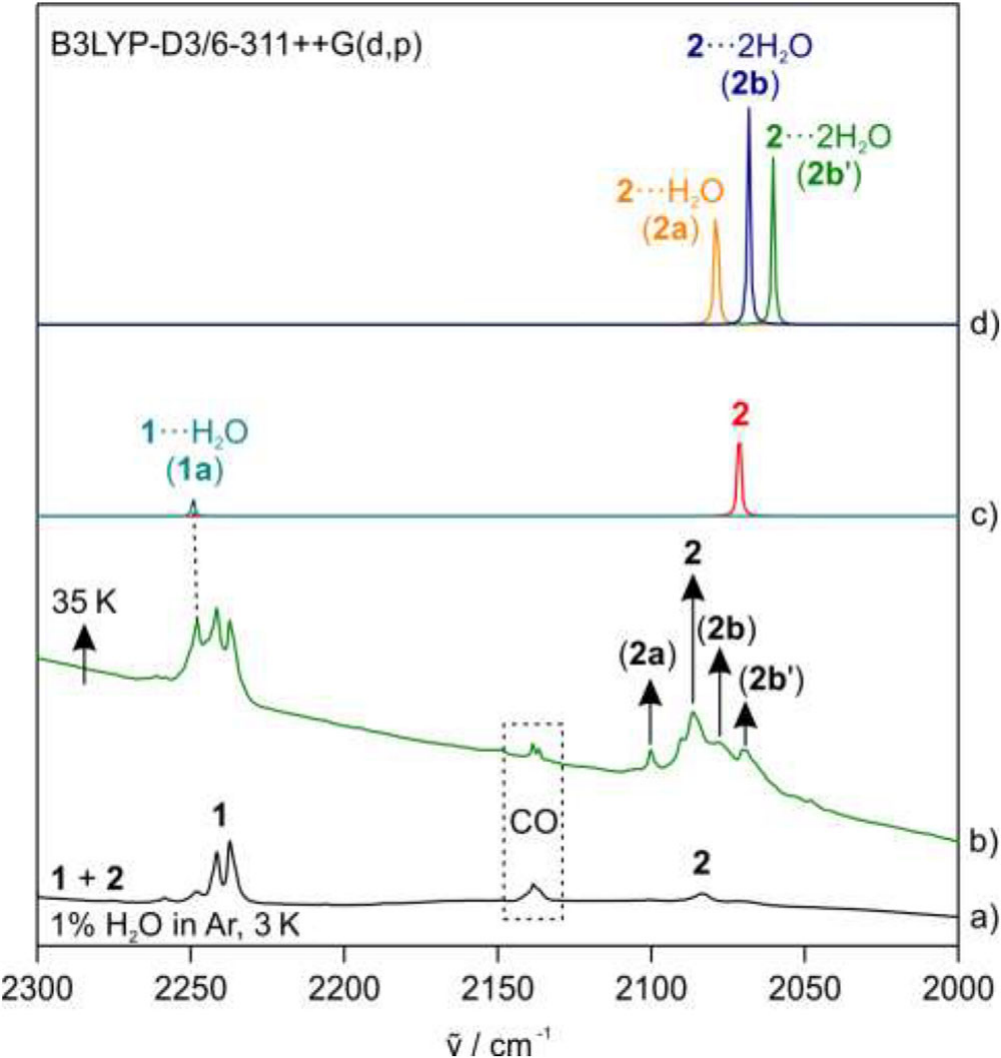
IR spectra showing the formation of various complexes of **1** and **2** with a single and two water molecules in an argon matrix doped with 1% water. a) **1** and **2** in an argon matrix doped with 1% water at 3 K. b) Subsequent annealing of the matrix at 35 K shows the formation of H‐bonded complexes. c) Computed CN stretching vibration band of radical anion **2** and **1a**, and d) **2a**, **2b**, and **2b'** at B3LYP‐D3/6–311++G(d,p) level of theory and scaled by a factor of 0.96.

The effect of local solvation of **2** was investigated by inducing interactions between matrix‐isolated molecules of **2** and a limited number of water molecules. This was achieved using argon matrices doped with 1% water, with the diffusion of water molecules controlled via the matrix temperature. After the codeposition of sodium and **1** in an argon matrix doped with water, we observed matrix‐isolated **1**, water monomers, some water dimers, and a small amount of **2** formed due to the reaction between sodium and **1**. The matrix was annealed to 35 K for several minutes, which allowed water molecules to diffuse and form aggregates and complexes. IR spectra were recorded after lowering the temperature of the matrix back to 3 K to stop diffusion. This annealing of the matrix resulted in the formation of new bands at 2248, 2100, 2078, and 2070 cm^−1^, which are assigned to hydrogen‐bonded complexes **1a** (**1**···H_2_O), **2a** (**2**···H_2_O), **2b** and, **2b'** (**2**···2H_2_O), respectively (Figure 2). The IR band assignments are confirmed by DFT calculations at the B3LYP‐D3/6–311++G (d,p) level of theory. The assignment was further verified by the isotopic shift observed for benzonitrile(nitrile‐^13^C)‐**2** in the CN stretching mode (Figure S4).

**Figure 3:**
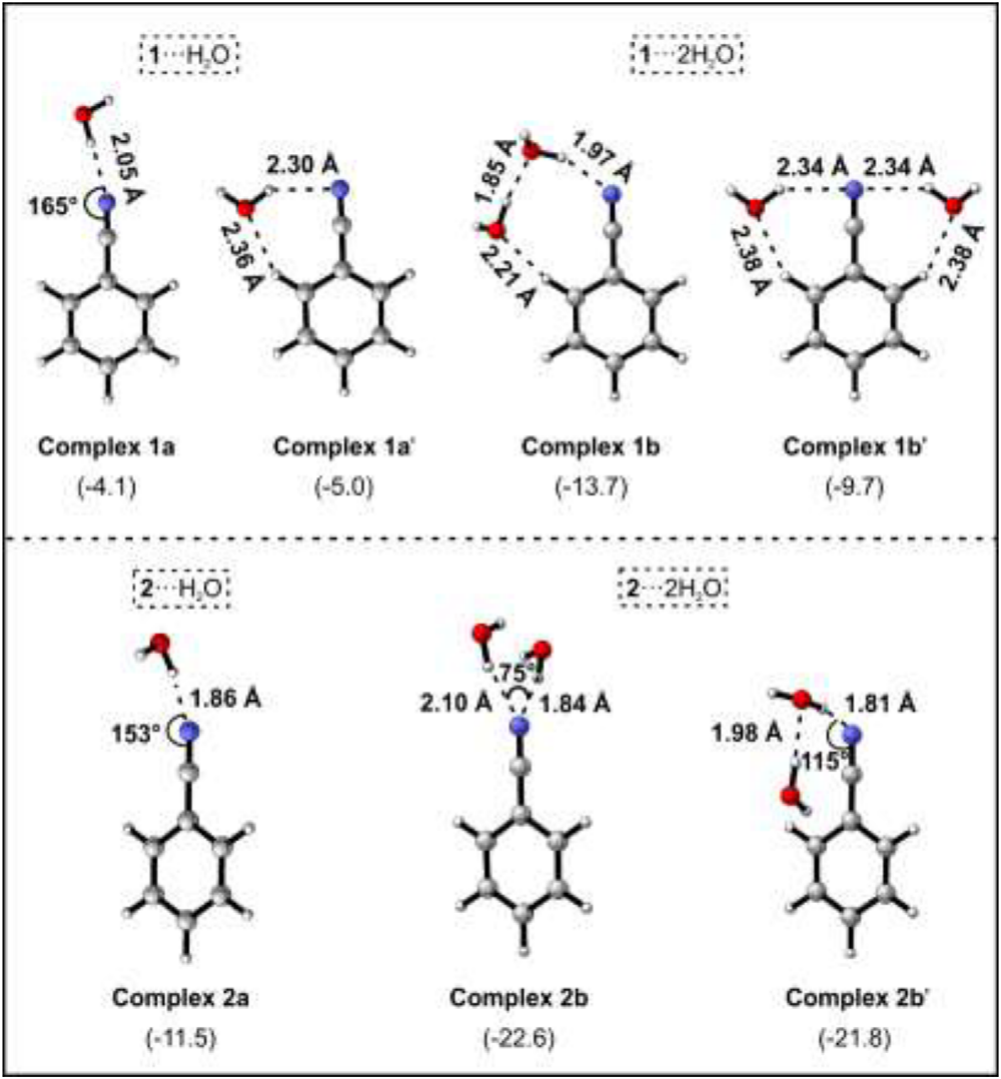
a) Optimized structure of the complexes of **1** and **2** with one and two water molecules at B3LYP‐D3/6–311++G(d,p) level of theory. Selected geometry parameters and ZPE‐corrected energies (kcal/mol) are shown.

The CN stretching vibration of complex **2a** shows an unusual blue shift of 17 cm^−1^ compared to **2** (Figure 2) due to the formation of a linear hydrogen‐bonded complex (Figure 3). This interaction leads to a contraction in the CN bond as observed in benzonitrile, which results in a blue shift of the IR band (Figure 2).^[^ ^22^, ^23^, ^24^, ^25^ ^]^ In contrast, radical anion **2** forms two complexes, **2b** and **2b'**, with two water molecules, as shown in Figure 3. This results in a red shift of 5 and 13 cm⁻¹, respectively, in the CN stretching vibration compared to that of **2** (Figure 2, Table 1).

**Table 1:** Experimental and calculated frequencies of the CN stretching vibration of **1**, **2**, and their corresponding water complexes.

		Experiments^[^ ^a^ ^]^		Calculated^[^ ^b^ ^]^	
Species		Argon	Water	Gas phase	Water
**1**	–	2241 (2187) 2238 (2184)	2235 (2179)	2333 (2278)	2320 (2265)
**1**···H_2_O	**1a**	2248 (2194)	–	2343 (2287)	–
**2**	–	2083 (2035)	2067 (2019)	2158 (2105)	2123 (2071)
**2**···H_2_O	**2a**	2100 (2049)	–	2166 (2112)	–
**2**···2H_2_O	**2b**	2078 (2026)	–	2155 (2101)	
	**2b'**	2070 (2020)	–	2146 (2093)	–

^[a]^ Experiments in an argon matrix doped with 1% of water and low‐density amorphous (LDA) water ice matrices.

^[b]^ Calculated in the gas phase and water PCM model with B3LYP‐D3/6–311++G(d,p) level of theory. Values in parentheses belong to the experiments performed using [^13^C‐**1**].

### 2.3 Computations of Complexes of **1** and **2** with Water Molecules

Complexes of **1** and **2** with water molecules have been extensively studied computationally by various research groups.^[^ ^15^, ^26^, ^27^ ^]^ The computations predict two complexes between **1** and a single water molecule. The more stable complex shows a cyclic structure **1a'** featuring interactions between the ortho‐hydrogen of **1** and the oxygen atom of water, while the hydrogen atom of water interacts with the nitrogen of **1** (Figure 3). The linear acyclic complex **1a** is stabilized by a linear O─H⋯N interaction between the nitrogen of **1** and the hydrogen atom of water. Complex **1a** is approximately 0.9 kcal/mol higher in energy than complex **1a'**. In various gas‐phase studies, exclusively complex **1a'** was identified.^[^ ^27^ ^]^ In contrast, in cryogenic matrices, only complex **1a** was found, indicating the strong effect of the matrix surroundings on complex formation.^[^ ^19^ ^]^ Krychako and coworkers investigated the complex between benzonitrile **1** and two water molecules computationally.^[^ ^26^ ^]^ The two most stable complexes are a one‐sided ring (**1b**, Figure 3) and a double‐sided one (**1b'**, Figure 3). Complex **1b**, which can also be identified as the complex of **1** with water dimer is calculated to be 4 kcal/mol more stable than complex **1b'**.

For the complex between radical anion **2** and a single water molecule, computations predict an acyclic structure as the most stable complex (Figure 3), which is stabilized by the high electron density at the CN group (Figure 3).^[^ ^15^ ^]^ Consequently, the O─H⋯N interaction distance decreases from 2.05 Å in complex **1a** to 1.86 Å in complex **2a**. The two complexes **2b** and **2b'**, between radical anion **2** and two water molecules are identified by gas‐phase calculations at the B3LYP‐D3/6–311++G(d,p) level of theory. Complex **2b** involves interactions of the two hydrogen atoms of two water molecules with the negatively charged nitrogen atom. In contrast, complex **2b'** exhibits an additional interaction of the hydrogen atom of the second water molecule and the Π‐cloud of the phenyl ring. Complex **2b** is predicted to be approximately 0.8 kcal/mol more stable than complex **2b'**. The complexation energies of **2b** (−22.6 kcal/mol) and **2b'** (−22.8 kcal/mol) predicted for **2**···2H_2_O are almost twice that of complex **2a** for **2**···H_2_O (−11.5 kcal/mol). Due to polarization, the water molecule in complex **1a** becomes a stronger H‐bond acceptor than monomeric water.^[^ ^28^ ^]^ Consequently, the energy gained by attachment of the second water molecule is higher than the dissociation energy of the water dimer (−3.16 kcal/mol),^[^ ^29^ ^]^ reflecting cooperativity effects similar to those observed in bulk water.^[^ ^30^ ^]^

### 2.4 Formation of Radical Anion 2 in Amorphous Water Ice

**Figure 4:**
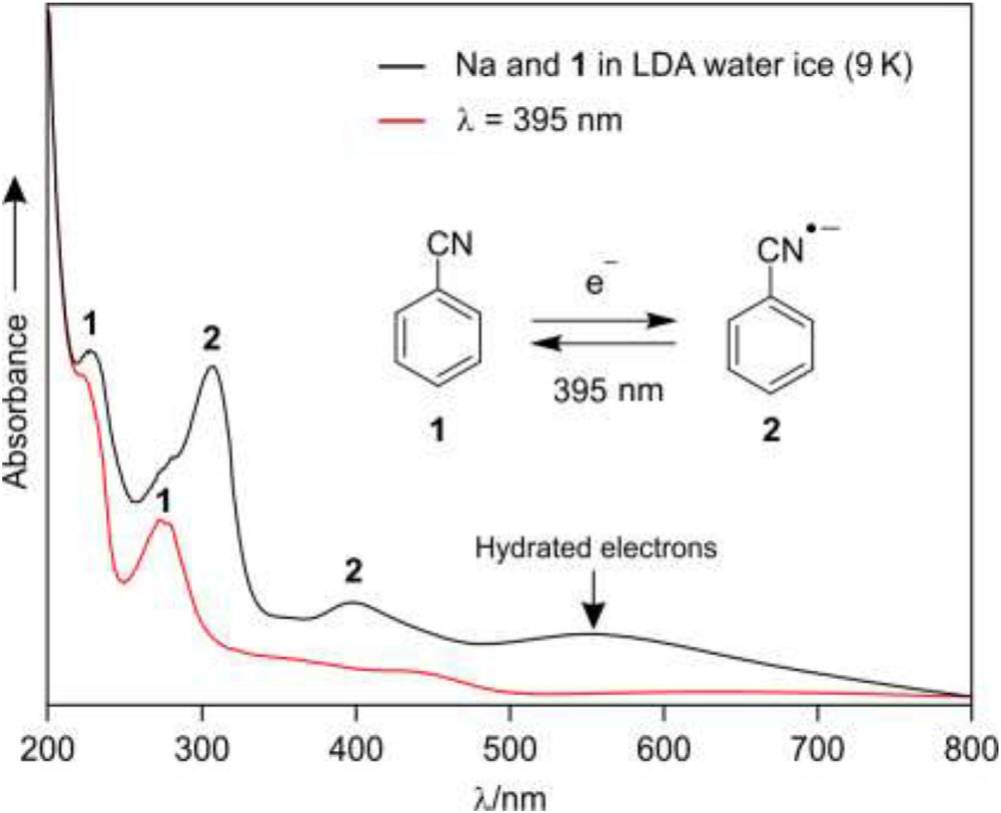
UV‐vis spectrum of **1** and sodium in LDA water ice at 9 K showing the formation of radical anion **2** (black line). Photolysis with 395 nm light results in a decrease of bands **2** and an increase of bands of **1** (red line).

**Figure 5:**
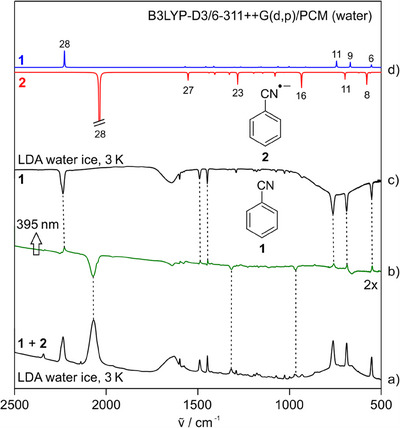
IR spectra showing reactions of **1** with sodium in LDA water ice at 3 K. (a) Spectrum obtained after codeposition of sodium and **1**. (b) Difference IR spectrum of the same matrix showing changes upon photolysis with 395 nm light. Bands pointing downward are assigned to **2**, and bands pointing upward are assigned to **1**. (c) IR spectrum of **1** at 3 K (multiplied by ‐1). (d) IR spectrum of **1** (pointing upward) and **2** (pointing downward) computed at the B3LYP‐D3/6–311 + G(d,p)/PCM (water) level of theory.

Upon deposition of sodium in LDA water ice matrices, sodium ionizes instantly, generating electrons that are subsequently trapped in the water matrix. These electrons are readily bleached by visible light irradiation.^[^ ^21^ ^]^ When sodium vapor and **1** are codeposited in LDA water ice matrices, some of the electrons react with **1** during deposition, leading to the formation of radical anion **2** (Figure 4). The generation of **2** is confirmed by absorption bands at 306 and 395 nm, which are in good agreement with the bands observed by Chutny and coworkers.^[^ ^12^ ^]^ In addition, bands associated with unreacted **1** and trapped electrons are also observed due to insufficient electron transfer. Irradiation with 395 nm light for an extended period of time leads to a decrease in the bands of **2** and an increase in the bands of **1**, indicating the photoionization of **2** under these conditions. The broad band associated with the hydrated electrons bleaches completely (Figure 4).^[^ ^21^ ^]^ The IR spectra obtained after the codeposition of sodium and **1** in LDA water show bands of the radical anion **2** together with that of the remaining **1**. The CN stretching vibration of **2** in LDA water ice is found at 2067 cm^−^^1^, red‐shifted by 16 cm^−1^ compared to argon (Figure 5, Table 1). This red shift is comparable to the 13 cm^−1^ observed for **2**···2H_2_O (Table 1). Irradiation of the matrix with 395 nm light leads to a slow decrease of **2** and an increase of **1** due to the photoionization of **2**. The ^13^C isotopic shift of the CN stretching vibration of radical anion **2** is 52 cm^−1^, in good agreement with the computed shift of 55 cm^−1^ (Figure S5).

The IR spectra obtained after the codeposition of sodium and **1** in LDA water show bands of the radical anion **2** together with that of the remaining **1**. The CN stretching vibration of **2** in LDA water ice is found at 2067 cm^−^^1^, red‐shifted by 16 cm^−1^ compared to argon (Figure 5). This red shift is comparable to the 13 cm^−1^ observed for **2**···2H_2_O. Irradiation of the matrix with 395 nm light leads to a slow decrease of **2** and an increase of **1** due to the photoionization of **2**. The ^13^C isotopic shift of the CN stretching vibration of radical anion **2** is 52 cm^−1^, in good agreement with the computed shift of 55 cm^−1^ (Figure S5).

In LDA water ice, the electron transfer is more efficient than in an argon matrix, resulting in a higher yield of **2**. This increased efficiency can be attributed to two main factors: First, the high concentration of hydrated electrons in the matrix, which reacts instantaneously with **1** to form the radical anion **2**. Second, computations using an implicit solvation model at the B3LYP‐D3/6–311++G(d,p) level of theory suggest that the EA of **1** significantly increases from 0.28 eV (6.4 kcal/mol) in the gas phase to 2.24 eV (51.6 kcal/mol) in water. The increased EA in LDA water ice results from the enhanced stability of **2** due to solvation.

## 3 Conclusion

The matrix isolation technique was used to generate the benzonitrile radical anion **2** by cocondensation of sodium and **1** in argon and LDA water ice matrices. In solid argon, sodium atoms can be isolated, and a small amount of radical anion **2** is formed during deposition. In contrast, sodium spontaneously dissociates into Na⁺ and hydrated electrons in the water matrix. The dissociation of sodium atoms in water is driven by the high EA of water ice and the stabilization of Na⁺ in the polar environment. The presence of hydrated electrons, along with the enhanced stability of radical anion **2** via solvation, leads to a higher yield of **2** compared to that observed in the argon matrix.

In argon matrices doped with 1% water, the influence of hydrogen bonding on the spectroscopic properties of **2** can be investigated. At such low concentrations, water only slightly increases the bulk polarity of the matrix, enabling the study of hydrogen bonding via diffusion experiments. After deposition at 3 K, these matrices contain mainly isolated molecules of **1**, **2**, and monomeric water molecules. When such water‐doped Ar matrices are annealed for several minutes at 35 K, water molecules diffuse in the solid argon, forming hydrogen‐bonded complexes between **1**, **2**, and water. Since this annealing process does not significantly alter the bulk polarity of the matrix, the effects of local solvation (formation of specific solvent − solute complexes) can be studied without changing the overall matrix polarity.

Hydrogen bonding between **2** and a single water molecule induces an unusual blue shift of approximately 17 cm^−1^ in the CN stretching vibration. In contrast, interaction with two water molecules results in the formation of two distinct complexes, **2b** and **2b'**, which exhibit red shifts of 5 cm^−1^ and 13 cm^−1^, respectively.

The study of the benzonitrile radical anion plays a key role in advancing our understanding of electron‐driven chemistry in both astrochemical and prebiotic environments. In interstellar space, benzonitrile can form its radical anion upon radiation exposure, potentially driving reactions that lead to the synthesis of prebiotic molecules, including amino acids and nucleobases, emphasizing its relevance to the origins of life. The hydrogen bonding between the benzonitrile radical anion and water molecules can greatly influence its reactivity, offering insights into solvent effects and the stabilization of charged species in aqueous environments—critical aspects in the study of biological redox processes and catalysis.

### 3.1 Experimental and Computational Details

Matrix isolation experiments were performed by standard techniques^[^ ^31^ ^]^ using a two‐staged closed‐cycle helium cryostat from Sumitomo Heavy Industries (3 K). Matrices were prepared by codeposition of benzonitrile **1** and sodium vapor along with an excess of gas onto a window held at 3 K. For the FTIR experiments, deposition was conducted on a CsI window with KBr external windows for the deposition chamber and a Bruker Vertex 70 spectrometer (0.5 cm^−1^) for recording and analyzing the spectra. In UV‐vis experiments, quartz windows were used for the external windows and sapphire for the deposition substrate, employing a Varian Cary 5000 spectrometer with 0.2 nm resolution. Compound **1** and its isotopomer ^13^C‐benzonitrile [^13^C]‐**1**, were obtained from Sigma‐Aldrich (99% purity) and subjected to several freeze‐pump‐thaw cycles before every experiment. H_2_O and D_2_O were used after several freeze‐pump‐thaw cycles too. Ar (99.9999%, Air Liquide) and LDA water ice matrices were prepared according to standard procedures.^[^ ^32^, ^33^ ^]^ Sodium was sublimed by placing a small cube of approximately 4 mm edge into a Knudsen cell and heating the cell resistively at temperatures ranging from 200 to 290 ⁰C. Electronic structure calculations were conducted using the Gaussian 09 software.^[^ ^34^ ^]^ Geometry optimization and vibrational frequencies were performed at the B3LYP level of theory coupled with the 6–311++G (d,p) basis set and using Grimme's dispersion correction GD3.^[^ ^35^, ^36^, ^37^ ^]^ Solvent effects for argon and water were considered implicitly by using the polarizable continuum model (PCM),^[^ ^38^ ^]^ but also explicitly by including one or two water molecules in the gas phase.

## Conflict of Interest

The authors declare no conflict of interest.

## Supporting information



Supporting Information

## Data Availability

The data that support the findings of this study are available from the corresponding author upon reasonable request.
