# Supplementary material for: Matrix Isolation and Solvation of the Benzonitrile Radical Anion
Source: Chemistry. 2025 May 30;31(35):e202501150. doi: 10.1002/chem.202501150 (PMC12188150; doi:10.1002/chem.202501150)
Supplement: Supplementary file 1 — Supporting Information [file CHEM-31-e202501150-s001.docx]

**Matrix Isolation and Solvation of the Benzonitrile Radical Anion**

Shubhra Sarkar, Ankit Somani, Wolfram Sander*^[a]^

Lehrstuhl für Organische Chemie II, Ruhr-Universität Bochum, 44801 Bochum, Germany

Content

Spectroscopic data

IR bands assignment

Cartesian coordinates of optimized structures

***Spectroscopic Data***

**
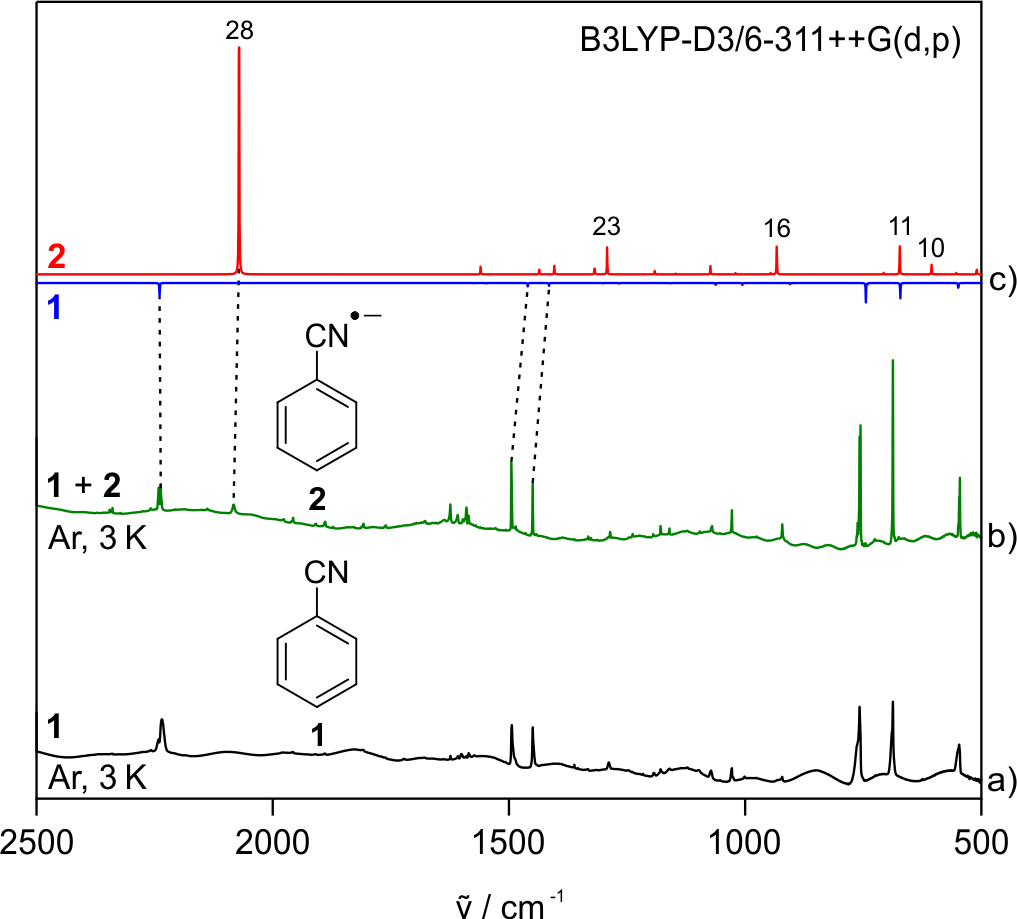
**

**Figure S1.** IR spectra showing the formation of benzonitrile radical anion **2** in an argon matrix at 3 K. a) IR spectrum of **1**. b) IR spectrum obtained after co-deposition of **1** and sodium, resulting in the formation of a small of radical anion **2**. c) Computed IR spectrum of **1** (pointing downwards) and **2** (pointing upwards) at B3LYP-D3/6-311++G(d,p) level of theory.


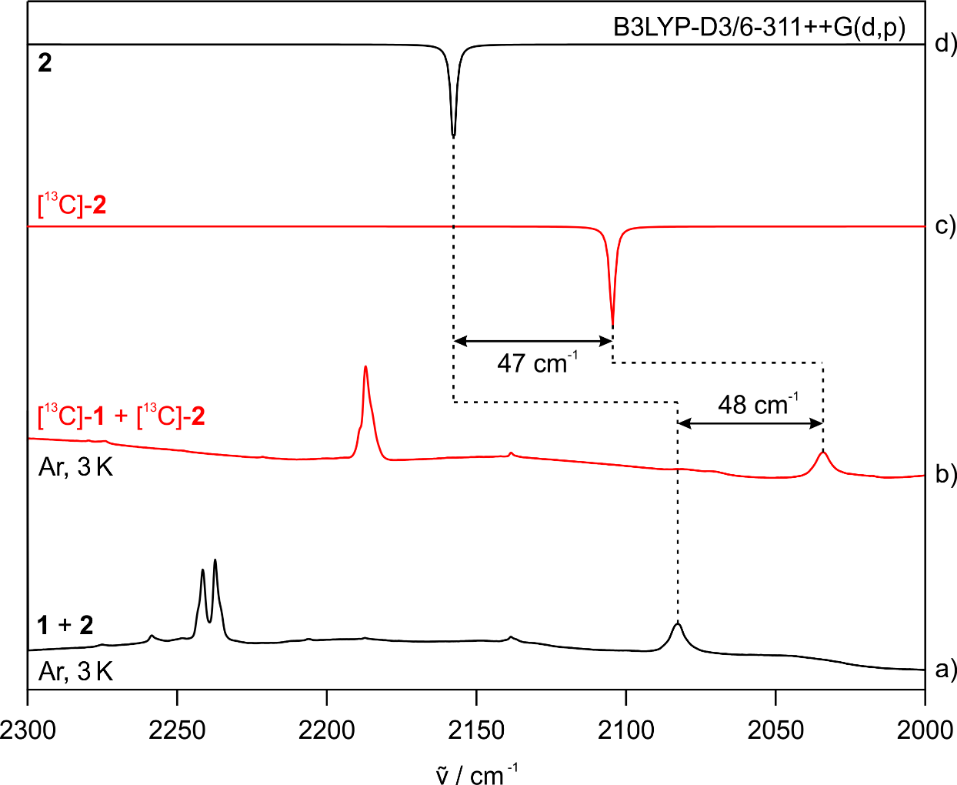


**Figure S2.** ^13^C isotopic shifts in the IR spectra of **1** and **2**. a) Experimental IR spectrum of [^12^C-**1**] and [^12^C-**2**] obtained after the co-deposition of **1** and sodium vapors in argon at 3 K. b) Experimental IR spectrum of isotopomers [^13^C-**1**] and [^13^C-**2**] obtained in a similar way. c) Calculated IR spectrum of radical anion [^13^C-**2**] and d) its corresponding isotopomer [^12^C-**2**] at B3LYP-D3/6-311++G(d,p) level of theory.


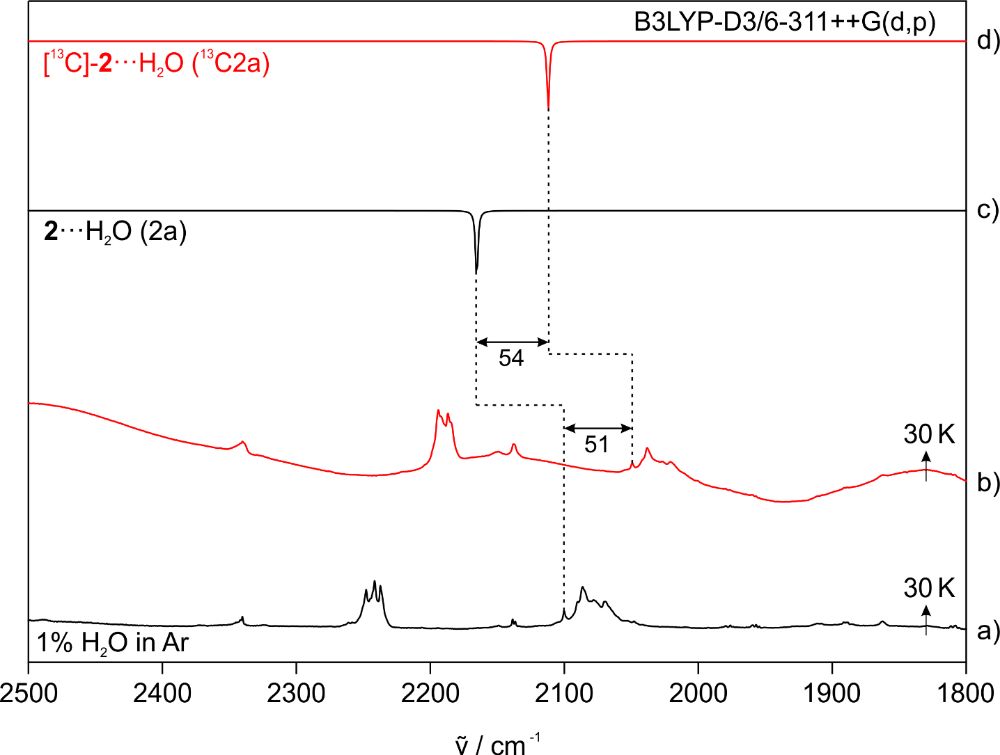


**Figure S3.** ^13^C isotopic shifts in the IR spectra of the CN stretching vibrational band of the complex **2a** (**2**···H_2_O). a) Experimental IR spectrum obtained after the co-deposition of **1** and sodium vapors in argon at 3 K and subsequently annealed to 30 K showing the formation of complex 2a. b) Experimental IR spectrum of isotopomers **^13^C2a** obtained similarly. c) Calculated IR spectrum of **2a** and d) its corresponding isotopomer **^13^C2a** at B3LYP-D3/6-311++G(d,p) level of theory.


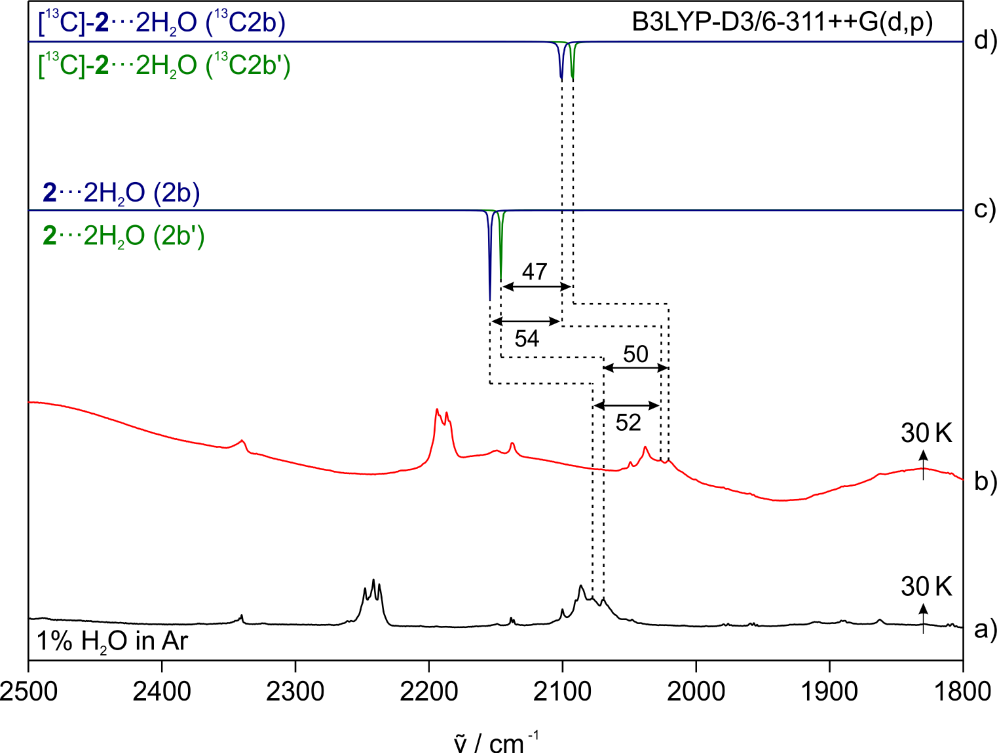


**Figure S4.** ^13^C isotopic shifts in the IR spectra of the CN stretching vibrational band of the complexes (**2**···2H_2_O) **2b** and **2b'**. a) Experimental IR spectrum of **2b**, and **2b'** obtained after the co-deposition of **1** and sodium vapors in argon at 3 K and subsequently annealed to 30 K. b) Experimental IR spectrum of isotopomers **^13^C2b**, and **^13^C2b'** obtained similarly. c) Calculated IR spectrum of **2b**, and **2b'** and d) its corresponding isotopomer **^13^C2b**, and **^13^C2b'** at B3LYP-D3/6-311++G(d,p) level of theory.


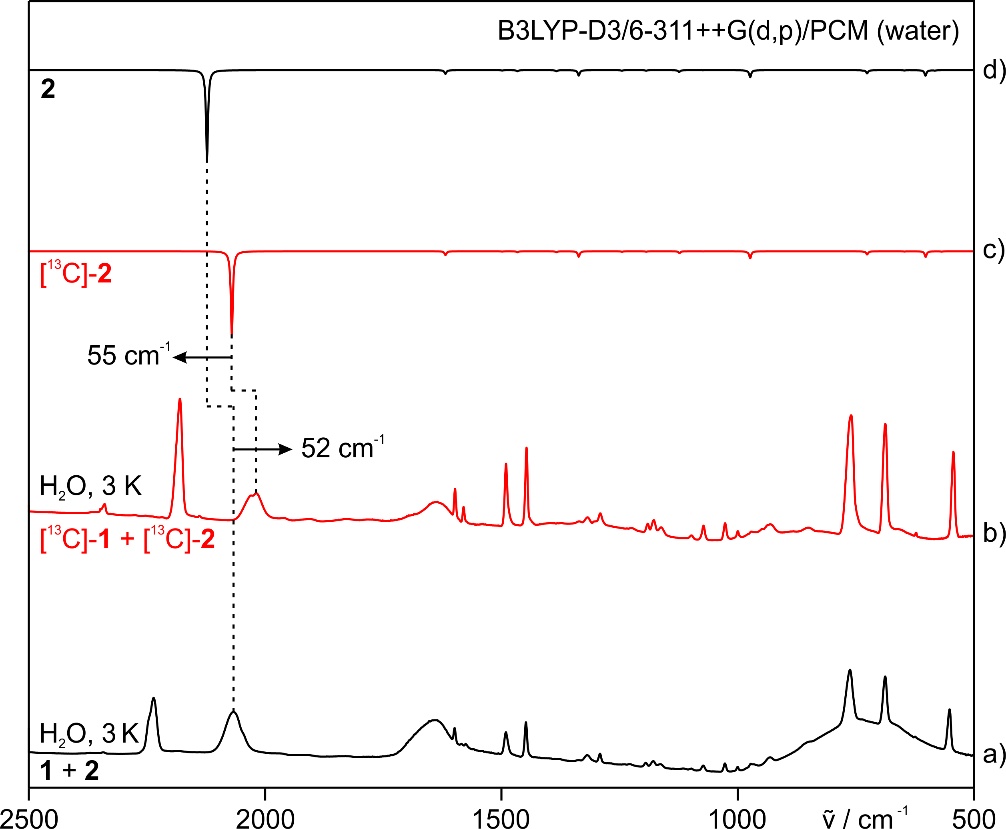


**Figure S5.** ^13^C isotopic shifts in the IR spectra of **1** and **2**. a) Experimental IR spectrum of [^12^C-**1**] and [^12^C-**2**] obtained after the co-deposition of **1** and sodium vapors in LDA water ice at 3 K. b) Experimental IR spectrum of isotopomers [^13^C-**1**] and [^13^C-**2**] obtained in a similar way. c) Calculated IR spectrum of radical anion [^13^C-**2**] and d) its corresponding isotopomer [^12^C-**2**] at B3LYP-D3/6-311++G(d,p)/PCM (water) level of theory.

***IR bands assignment***

**Table S1.** Experimental and calculated IR vibrational frequencies of Benzonitrile **1**.

| Calculated^[a]^ | | | |  | Water^[b]^ | Assignment |
| --- | --- | --- | --- | --- | --- | --- |
| Mode | ṽ  (cm^-1^) | I_abs_  (km/mol) | I_rel_  (km/mol) |  | ṽ  (cm^-1^) |  |
| 6 | 577 | 31 | 0.16 |  | 552 | CH wagging |
| 9 | 697 | 57 | 0.30 |  | 688 | CH wagging |
| 11 | 775 | 70 | 0.37 |  | 763 | CH wagging |
| 24 | 1472 | 13 | 0.07 |  | 1449 | In plane CH bending |
| 25 | 1518 | 9 | 0.05 |  | 1491 | In plane CH bending, CC str. |
| 27 | 1635 | 10 | 0.05 |  | 1600 | CC str. |
| 28 | 2320 | 191 | 1.00 |  | 2236 | CN str. |

^[a]^ Calculated at B3LYP-D3/6-311++G(d,p) level of theory.

^[b]^ LDA water ice matrix at 3 K.

**Table S2.** Experimental and calculated PCM (water) IR vibrational frequencies of Benzonitrile radical anion **2**.

| Calculated^[a]^ | | | |  | Water^[b]^ | Assignment |
| --- | --- | --- | --- | --- | --- | --- |
| Mode | ṽ  (cm^-1^) | I_abs_  (km/mol) | I_rel_  (km/mol) |  | ṽ  (cm^-1^) |  |
| 16 | 974 | 173 | 0.08 |  | 973 | Ring breathing |
| 19 | 1124 | 44 | 0.02 |  | 1114 | In plane CH bending |
| 20 | 1194 | 11 | 0.01 |  | 1170 | In plane CH bending |
| 21 | 1246 | 11 | 0.01 |  | 1228 | CCN str., In plane CH bending, Ring def. |
| 23 | 1337 | 133 | 0.06 |  | 1318 | CC str. |
| 24 | 1384 | 24 | 0.01 |  | 1376 | In plane CH bending |
| 25 | 1466 | 25 | 0.01 |  | 1425 | In plane CH bending, CCstr. |
| 27 | 1619 | 90 | 0.04 |  | 1576 | CC str. |
| 28 | 2123 | 2175 | 1.00 |  | 2076 | CN str. |

^[a]^ Calculated at B3LYP-D3/6-311++G(d,p)/PCM (water) level of theory.

^[b]^ LDA water ice matrix at 3 K.

***Cartesian Coordinates***

**Table S3.** Cartesian coordinates of optimized geometries of **1**, and **2** in gas phase at B3LYP-D3/6-31++G(d,p) level of theory.

| **1** | **2** |
| --- | --- |
| C 2.17625500 0.00000000 0.00000000  C 1.48056800 -1.20907100 0.00000000  C 0.08988700 -1.21563000 0.00000000  C -0.60996900 0.00000000 0.00000000  C 0.08988700 1.21563000 0.00000000  C 1.48056800 1.20907000 0.00000000  H 3.26025700 0.00000000 0.00000000  H 2.02162200 -2.14804700 0.00000000  H -0.45840100 -2.14956200 0.00000000  H -0.45840100 2.14956200 0.00000000  H 2.02162300 2.14804700 0.00000000  C -2.04160800 0.00000000 0.00000000  N -3.19717500 0.00000000 0.00000000 | C 2.21679600 0.00000100 -0.00000100  C 1.48117400 -1.21629800 0.00000000  C 0.10475600 -1.23234200 0.00000100  C -0.65507900 -0.00000100 0.00000000  C 0.10475500 1.23234200 0.00000000  C 1.48117300 1.21629800 0.00000000  H 3.30082400 0.00000100 -0.00000100  H 2.01646600 -2.16369000 0.00000000  H -0.43025000 -2.17697300 0.00000100  H -0.43025200 2.17697200 0.00000100  H 2.01646400 2.16369100 0.00000000  C -2.04850800 -0.00000100 0.00000000  N -3.22623600 0.00000100 0.00000000 |
| **E** + **ZPE =** -324.48589 H | **E** + **ZPE =** -324.49607 H |

**Table S4.** Cartesian coordinates of optimized geometries of complex **1a**, **1a'**, **1b**, **1b'**, **2a**, **2b**, and **2b'** at B3LYP-D3/6-31++G(d,p) level of theory.

| **1···H_2_O (1a)** | 1···H_2_O (1a') |
| --- | --- |
| C -3.12021200 -0.14102700 -0.00288500  C -2.33237700 -1.29237300 -0.00003200  C -0.94578900 -1.19048900 0.00253000  C -0.34566500 0.07739100 0.00215800  C -1.13682500 1.23560900 -0.00071900  C -2.52229300 1.11946700 -0.00324100  H -4.20072500 -0.22609200 -0.00487600  H -2.79810900 -2.27064800 0.00018500  H -0.32546700 -2.07818700 0.00469800  H -0.66345500 2.20966900 -0.00106400  H -3.13548300 2.01274900 -0.00551900  C 1.07999400 0.18742700 0.00459000  N 2.23104000 0.27225100 0.00661900  O 5.19983600 -0.25693800 -0.01065400  H 4.24587000 -0.08916000 -0.00743000  H 5.60040500 0.61538000 0.03849400 | C -2.52669000 0.78645700 0.00095400  C -1.34311400 1.52510600 -0.00153700  C -0.11080800 0.87996300 -0.00118400  C -0.07468600 -0.52336800 0.00141900  C -1.26223400 -1.26950500 0.00387100  C -2.48527300 -0.60834000 0.00377700  H -3.48277600 1.29748100 0.00040500  H -1.37856500 2.60819600 -0.00430900  H 0.82026900 1.43446500 -0.00233600  H -1.21836200 -2.35176100 0.00547900  H -3.40539000 -1.18069400 0.00552500  C 1.19191800 -1.18765000 0.00082900  N 2.23168700 -1.69310500 -0.00000900  O 3.17852500 1.30135700 -0.04559300  H 3.30997200 0.34440900 -0.04274300  H 3.97015600 1.67280600 0.35401300 |
| **E** + **ZPE =** -400.92975 H | **E** + **ZPE =** -400.93108 H |

| **1···2H_2_O (1b)** | **1···2H_2_O (1b')** |
| --- | --- |
| C 3.09665000 0.78567200 -0.04370900  C 1.89361900 1.49175800 -0.05884500  C 0.67775000 0.81595300 -0.02793300  C 0.68332500 -0.58708800 0.01700000  C 1.89006900 -1.30269700 0.03255600  C 3.09433000 -0.60938000 0.00235000  H 4.03855600 1.32199400 -0.06802200  H 1.89942000 2.57481300 -0.09521800  H -0.26806300 1.34833800 -0.03527100  H 1.87551200 -2.38524500 0.06757000  H 4.02986100 -1.15601400 0.01422500  C -0.56312900 -1.28589400 0.04330200  N -1.58011400 -1.83357000 0.05943100  O -4.03893000 -0.32356200 -0.03730500  H -4.57792600 -0.54526700 -0.80232500  H -3.37557900 -1.03211100 0.03219400  O -2.38768300 1.95984600 0.03873600  H -2.76791200 2.55385300 0.69171600  H -3.01585800 1.21440700 -0.02066200 | C 2.91566500 0.00185500 -0.00242800  C 2.22284700 -1.20963700 -0.00201100  C 0.83233300 -1.22272900 -0.00174500  C 0.14176700 -0.00060700 -0.00188000  C 0.83017700 1.22270000 -0.00262900  C 2.22078500 1.21208100 -0.00281500  H 3.99991500 0.00271800 -0.00187100  H 2.76644400 -2.14711400 -0.00092600  H 0.27243200 -2.15045600 -0.00124600  H 0.26863300 2.14943800 -0.00284400  H 2.76263400 2.15057100 -0.00232300  C -1.28685900 -0.00159600 0.00014900  N -2.44386500 -0.00240800 0.00314600  O -1.90086000 -3.11478200 0.04381200  H -2.41849600 -3.81370700 -0.36603400  H -2.47041400 -2.33511100 0.04432900  O -1.90456800 3.11529200 0.04618000  H -2.41677700 3.80836800 -0.38016500  H -2.47418500 2.33566900 0.04926700 |
| **E** + **ZPE =** -477.3822 H | **E** + **ZPE =** -477.37592 H |

| **2···H_2_O (2a)** |
| --- |
| C -3.07620800 0.00014800 0.26204800  C -2.35272400 1.21562500 0.14530700  C -2.35283900 -1.21545400 0.14586400  H -2.88191100 2.16224300 0.22949400  H -2.88211800 -2.16198200 0.23048700  C -0.99374400 1.23324600 -0.07280700  C -0.99386200 -1.23330400 -0.07224100  H -0.46543900 2.17729500 -0.15861200  H -0.46564400 -2.17744100 -0.15760800  C -0.24527700 -0.00009100 -0.19222000  H -4.14642000 0.00023600 0.43329200  C 1.12567400 -0.00020700 -0.40785600  N 2.28910500 -0.00032200 -0.59688300  O 4.92759900 0.00014800 0.42850500  H 4.67500900 0.00104900 1.35577800  H 4.05587700 -0.00010700 -0.03126000 |
| **E** + **ZPE =** -400.95171 H |

| **2···2H_2_O (2b)** | **2···2H_2_O (2b')** |
| --- | --- |
| C -3.71989100 0.09577000 -0.02565200  C -3.05475300 -1.15584500 -0.00508100  C -1.68180100 -1.24700400 0.03217800  C -0.86162500 -0.05500900 0.05154700  C -1.55224000 1.21676800 0.03222000  C -2.92733900 1.27039700 -0.00521100  H -4.80172800 0.15264500 -0.05411200  H -3.64024200 -2.07205900 -0.01865900  H -1.19695700 -2.21736300 0.04758700  H -0.96843200 2.13109200 0.05001800  H -3.41343300 2.24305000 -0.01810500  C 0.51900000 -0.12580400 0.08382400  N 1.69939300 -0.18306500 0.11430700  O 4.36984700 0.09198100 1.44951500  H 3.40847000 -0.00172900 1.33295400  H 4.67151600 0.04805600 0.53148500  O 3.97166300 -0.04633600 -1.53315900  H 3.86282300 0.81785200 -1.93994900  H 3.12205700 -0.18089300 -1.04517600 | C 3.04436400 0.46897700 0.06643800  C 2.39816800 0.23795000 -1.17404600  C 1.13871900 -0.31187500 -1.24451100  C 0.41961700 -0.67105400 -0.03942900  C 1.09119600 -0.43070500 1.22139400  C 2.35174600 0.12073400 1.25282000  H 4.03369700 0.90923700 0.10671400  H 2.90529200 0.50431500 -2.09816200  H 0.66305700 -0.47328500 -2.20588900  H 0.58037600 -0.68474800 2.14410000  H 2.82285700 0.29516400 2.21713800  C -0.83981100 -1.24581500 -0.09049100  N -1.92542000 -1.71189100 -0.13024700  O -3.88928400 0.26123600 0.02827700  H -3.28081900 -0.51229100 -0.09883100  H -4.12272700 0.21679000 0.96015000  O -1.66138200 2.18199300 -0.02281900  H -2.43490800 1.59548000 -0.08612300  H -0.90754500 1.57746500 -0.02408100 |
| **E** + **ZPE =** -477.40665 H | **E + ZPE =** -477.40534 H |

**Table S5.** Cartesian coordinates of optimized geometries of **1**, and **2** with the inclusion of water as solvent B3LYP-D3/6-31++G(d,p) level of theory.

| **1** | **2** |
| --- | --- |
| C 0.00000000 0.00000000 -2.17381800  C 0.00000000 1.21097300 -1.47907000  C 0.00000000 1.21874600 -0.08867300  C 0.00000000 0.00000000 0.60813000  C 0.00000000 -1.21874600 -0.08867300  C 0.00000000 -1.21097300 -1.47907000  H 0.00000000 0.00000000 -3.25745800  H 0.00000000 2.14966000 -2.01975200  H 0.00000000 2.15391800 0.45748700  H 0.00000000 -2.15391800 0.45748700  H 0.00000000 -2.14966000 -2.01975200  C 0.00000000 0.00000000 2.03805900  N 0.00000000 0.00000000 3.19438400 | C -2.21142100 0.00000100 0.00000000  C -1.48061900 -1.21725000 0.00000000  C -0.10308100 -1.23660600 0.00000000  C 0.65465900 -0.00000100 0.00000000  C -0.10308000 1.23660500 0.00000000  C -1.48061800 1.21725100 0.00000000  H -3.29460400 0.00000100 0.00000100  H -2.01748000 -2.16217700 0.00000000  H 0.42908300 -2.18234000 0.00000000  H 0.42908500 2.18233900 0.00000000  H -2.01747800 2.16217900 0.00000000  C 2.04184800 -0.00000100 0.00000000  N 3.22360800 0.00000100 0.00000000 |
| **E** + **ZPE =** -324.49415 H | **E** + **ZPE =** -324.5764 H |
